# Ventriculitis Presenting as a Complication of Haemophilus Influenzae Mastoiditis and Meningitis

**DOI:** 10.7759/cureus.24480

**Published:** 2022-04-25

**Authors:** Amy Kiamos, Swetha R Nuthulaganti, Rahul Gujarathi, Narsimha Candula

**Affiliations:** 1 Internal Medicine, University of Florida College of Medicine – Jacksonville, Jacksonville, USA; 2 Hospital Medicine, University of Florida Health, Jacksonville, USA

**Keywords:** mastoiditis, ventriculoperitoneal shunt, meningitis, ventriculitis, haemophilus influenzae

## Abstract

Ventriculitis is a rare intracranial disease with potentially life-threatening consequences. Here, we present a case of acute mastoiditis that progressed to *Haemophilus influenzae *meningitis evolving to ventriculitis. This case was complicated by hydrocephalus that subsequently required the placement of a ventriculoperitoneal shunt. In patients presenting with mastoiditis, it is imperative to initiate early and appropriate treatment to prevent disease progression and devastating outcomes. We aim to increase recognition of potential complications and encourage childhood vaccination of *Haemophilus influenzae*.

## Introduction

*Haemophilus (H.) influenzae* is a gram-negative organism that is a primary colonizer of the respiratory tract. Encapsulated strains tend to cause more invasive diseases compared to unencapsulated strains [1]. *H. influenzae* type B (Hib) accounts for 95% of invasive diseases in children, and current guidelines only recommend vaccination for Hib in children under five years old [2]. Hib is commonly associated with invasive diseases such as bacteremia, meningitis, pneumonia, and epiglottitis. Our case demonstrates a rare case of ventriculitis secondary to the progression of acute mastoiditis and meningitis. It is important to be aware of the central nervous system complications of *H. influenzae,* as early recognition and prompt treatment can prevent the extension of the disease and lead to better outcomes.

## Case presentation

A 65-year-old male with a medical history significant for chronic obstructive pulmonary disease (COPD) presented to the hospital with new-onset altered mental status. For one week prior to presentation, the patient endorsed experiencing a headache and ear pain. The patient was unable to give further history due to his waxing and waning mentation. The remainder of his history was provided by the patient’s family. Reportedly, the patient did not have any recent sick contacts, travel, or history of alcohol or substance use. The patient’s vaccinations were up to date. The patient’s initial vital signs were notable for a rectal temperature of 101.1°F, a pulse rate of 160 beats per minute, a blood pressure of 167/144 mmHg, a respiratory rate of 24 breaths per minute, and oxygenation saturation of 98% on room air. His physical exam was significant for erythematous tympanic membranes bilaterally, with right greater than left. He was agitated, oriented to self only, and answered most questions nonsensically. There was no nuchal rigidity or focal neurological deficits noted. Subsequently, he was admitted to the intensive care unit (ICU) for concern of sepsis with acute encephalopathy. He was started on vancomycin, cefepime, and acyclovir empirically.

**Figure 1:**
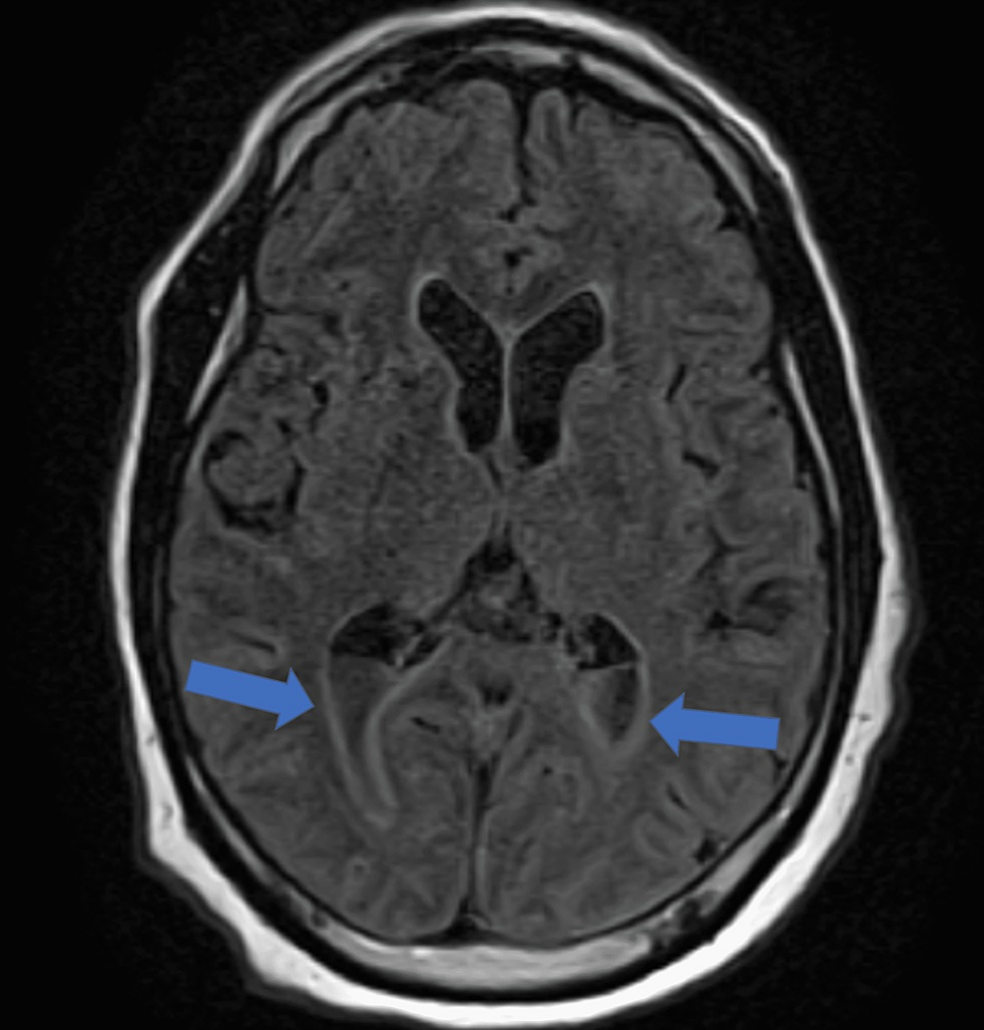
MRI of the brain demonstrating ventriculitis with thickening of the subependymal lining along the lateral ventricles and pus layering within the ventricular system

Laboratory results were significant for white blood cells (WBCs) of 11.62 thousand/mm^3^ with 21.1% bandemia and lactic acid of 6.9 mmol/L. The patient’s electrolytes, ammonia, and TSH were unremarkable. Blood cultures grew *Haemophilus influenzae*. Computerized tomography (CT) of the head demonstrated a right mastoid effusion concerning for mastoiditis. Lumbar puncture was consistent with bacterial meningitis with very low glucose of 4 mg/dL, high protein of 415 mg/dL, and WBCs of 2,520 UL with 95% neutrophils. The patient continued to have worsening mentation and fevers even after receiving antibiotics for a couple of days and further imaging was obtained. Magnetic resonance imaging (MRI) of the brain demonstrated ventriculitis with enhancement and thickening of the subependymal lining along the lateral ventricles and pus layering within the ventricular system (Figure 1). MRI further confirmed meningitis of the left hemisphere along with severe right mastoid and right middle ear effusions. Antibiotic coverage was extended to cover anaerobes. Otolaryngology then performed a myringotomy with tube placement.

**Figure 2:**
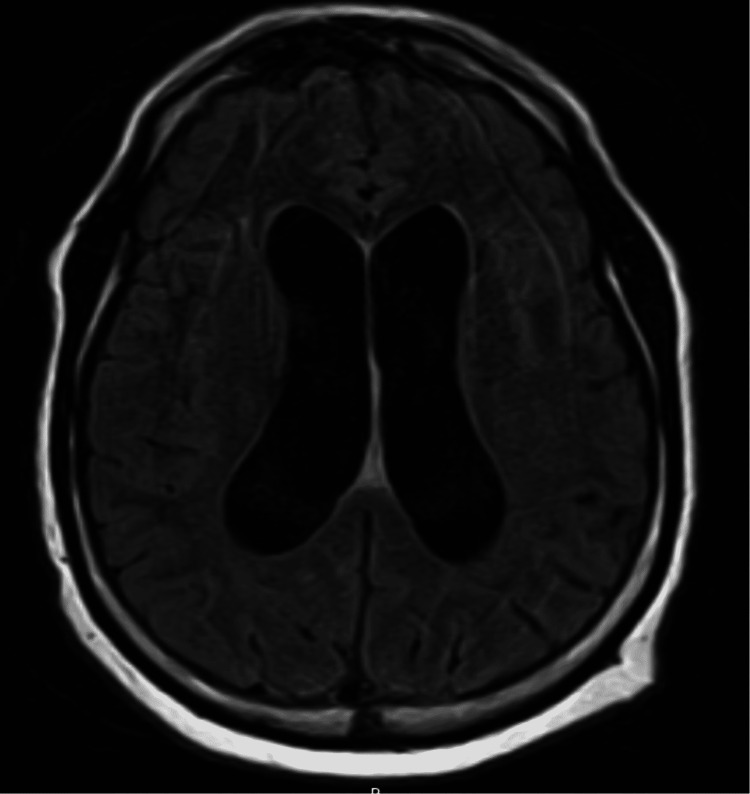
MRI of the brain demonstrating moderate ventricular dilation concerning for communicating hydrocephalus

Following a couple of days, the patient's altered mentation eventually improved; however, he continued to experience low-grade fevers and worsening headaches. A repeat MRI of the brain showed an interval increase in dilation of the ventricular system with communicating hydrocephalus and increased thickness of the subependymal lining of lateral ventricles compatible with ventriculitis (Figure 2). The patient underwent serial lumbar punctures and a lumbar drain was placed. Neurosurgery placed a ventriculoperitoneal shunt for further treatment of hydrocephalus. He completed a six-week course of ceftriaxone and metronidazole and was discharged to home. He maintained appropriate follow-up and had complete resolution of infection without any neurological deficits.

## Discussion

*Haemophilus influenzae* is a gram-negative coccobacillus that is predominantly found in the human respiratory tract. *H. influenzae* strains are subdivided into two categories, encapsulated (typable) and unencapsulated (non-typeable) [1]. Encapsulated strains have six different serotypes of capsular polysaccharides, ranging from a-f. Encapsulated strains tend to cause more invasive diseases, including bacteremia, meningitis, pneumonia, and epiglottitis. *H. influenzae* type B (Hib) is the most virulent strain, accounting for 95% of invasive diseases in children [2]. Non-typeable *H. influenzae* strains tend to be less invasive, causing common infections such as otitis media, sinusitis, and conjunctivitis [3].

Invasive Hib infections have greatly decreased after the introduction of the polysaccharide conjugate vaccine in industrialized countries [4]. The vaccines contain the type b capsular polysaccharide causing protection against Hib infections. Patients with asplenia are at risk for infections with encapsulated organisms and rely on this vaccine for protection against Hib; however, the vaccine does not protect against non-typeable *H. influenzae* strains. There remains a high prevalence of invasive infections in underdeveloped countries where access to vaccination is limited [2]. However, recent studies have shown an increased incidence of invasive diseases caused by *H. influenzae* in adults primarily aged over 65 in the United States [5-6]. This could be due to waning immunity due to the absence of booster vaccines, decreased childhood vaccination, or the presence of other comorbidities [4,6]. Current guidelines recommend vaccination only for children less than five years of age. Further studies are needed to determine the need for Hib vaccinations in adults.

Ventriculitis is a rare intracranial disease defined by inflammation of the ependymal lining of the ventricles in the brain. Ventriculitis has a poor prognosis, with high in-hospital mortality rates. Most cases of ventriculitis are usually secondary to bacterial meningitis, brain abscesses, trauma, or device-related infections [7]. Ventriculitis has also been noted to result from a complication of an otological infection, specifically mastoiditis secondary to acute otitis media [8]. Acute mastoiditis is a severe intra-temporal complication of acute otitis media. If not treated correctly, the infection can extend from the mastoid cells leading to complications such as meningitis, abscesses, and vascular thrombosis [9]. It is important to initiate early and appropriate treatment for mastoiditis to prevent life-threatening consequences of the extension of infection. Most patients with intracranial complications present with continued headaches, fevers, vomiting, seizures, lethargy, or cranial nerve abnormalities [10]. In cases of ventriculitis, intraventricular pus layering should prompt careful monitoring for hydrocephalus [7].

Ventriculitis as a complication of *H. influenzae* is exceedingly rare. Only a few cases have been reported in children and in those who have undergone neurosurgical procedures [11]. Our case is unique, as our patient was an older adult with a relatively insignificant past medical and surgical history. He had no prior neurosurgical interventions prior to being diagnosed with ventriculitis.

## Conclusions

This case highlights a rare presentation of ventriculitis secondary to acute mastoiditis progressing to Hib meningitis in an adult. It is important to initiate early and appropriate treatment for acute mastoiditis to prevent the extension of the disease and devastating outcomes. Our goal is to increase awareness of monitoring for these complications and to encourage vaccination against *H. influenzae*.
